# Rapid Nontranscriptional Effects of Calcifediol and Calcitriol

**DOI:** 10.3390/nu14061291

**Published:** 2022-03-18

**Authors:** Simone Donati, Gaia Palmini, Cinzia Aurilia, Irene Falsetti, Francesca Miglietta, Teresa Iantomasi, Maria Luisa Brandi

**Affiliations:** 1 Department of Experimental and Clinical Biomedical Sciences, University of Florence, 50139 Florence, Italy; simone.donati@unifi.it (S.D.); gaia.palmini@unifi.it (G.P.); cinzia.aurilia@unifi.it (C.A.); irene.falsetti@unifi.it (I.F.); francesca.miglietta@unifi.it (F.M.); teresa.iantomasi@unifi.it (T.I.); 2 Fondazione Italiana Ricerca sulle Malattie dell’Osso (F.I.R.M.O. Onlus) Italian Foundation for the Research on Bone Diseases, 50141 Florence, Italy

**Keywords:** vitamin D, calcitriol, calcifediol, non-genomic actions, vitamin D receptor, membrane-associated rapid response to steroid

## Abstract

Classically, a secosteroid hormone, vitamin D, has been implicated in calcium and phosphate homeostasis and has been associated with the pathogenesis of rickets and osteomalacia in patients with severe nutritional vitamin D deficiency. The spectrum of known vitamin D-mediated effects has been expanded in recent years. However, the mechanisms of how exactly this hormone elicits its biological function are still not fully understood. The interaction of this metabolite with the vitamin D receptor (VDR) and, subsequently, with the vitamin D-responsive element in the region of specific target genes leading to the transcription of genes whose protein products are involved in the traditional function of calcitriol (known as genomic actions). Moreover, in addition to these transcription-dependent mechanisms, it has been recognized that the biologically active form of vitamin D_3_, as well as its immediate precursor metabolite, calcifediol, initiate rapid, non-genomic actions through the membrane receptors that are bound as described for other steroid hormones. So far, among the best candidates responsible for mediating rapid membrane response to vitamin D metabolites are membrane-associated VDR (VDRm) and protein disulfide isomerase family A member 3 (Pdia3). The purpose of this paper is to provide an overview of the rapid, non-genomic effects of calcifediol and calcitriol, whose elucidation could improve the understanding of the vitamin D_3_ endocrine system. This will contribute to a better recognition of the physiological acute functions of vitamin D_3_, and it could lead to the identification of novel therapeutic targets able to modulate these actions.

## 1. Introduction

Calcitriol (1α,25-dihydroxyvitamin D_3_ or 1α,25(OH)_2_D_3_), the biologically active form of vitamin D_3_, is a sterol hormone that is involved in the regulation of phosphate and plasma-ionized calcium levels by modulating their renal excretion, intestinal absorption, and calcium bone mobilization [1].

When low serum calcium concentrations are detected through the calcium-sensing receptor (CaSR) on the parathyroid glands, a G-protein coupled receptor, parathyroid hormone (PTH) secretion is triggered, thus activating calcitriol synthesis [2].

In addition to repressing parathyroid gene expression and cell proliferation by interacting with the vitamin D receptor (VDR), 1α,25(OH)_2_D_3_ maintains ideal serum calcium levels through its direct actions on the gut, the kidney, and the bone tissues [3].

**Figure 1:**
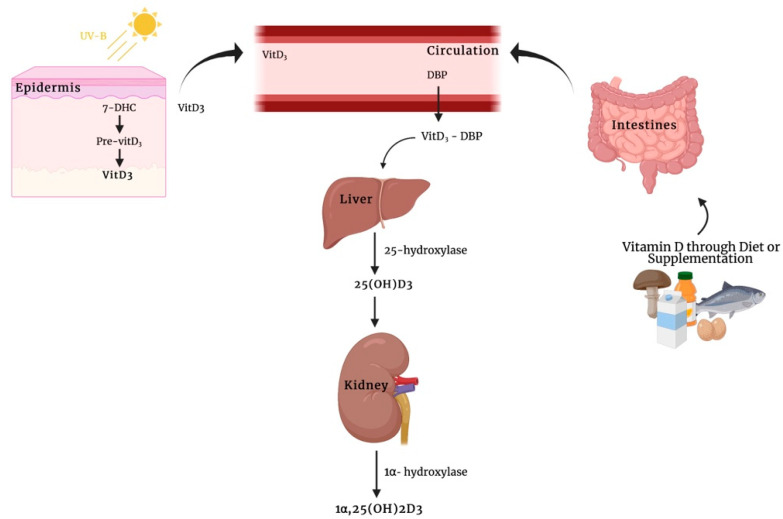
Schematic representation for the classical synthesis pathway of the biological active form of vitamin D_3_, 1α,25(OH)_2_D_3_. 7-DHC: 7-dehydrocholesterol; DBP: vitamin D binding protein. Image created by BioRender (https://app.biorender.com (accessed on 14 February 2022)).

Vitamin D_3_ is typically produced in the skin, where provitamin D_3_ (7-dehydrocholesterol (7-DHC)) is transformed into previtamin D_3_ by the exposure to ultraviolet B (UVB) radiation, which then undergoes a thermal isomerization to produce vitamin D_3_ [4] (Figure 1). Subsequently, vitamin D_3_ enters the bloodstream and reaches the liver where it is promptly hydroxylated by the 25-hydroxylase, a member of cytochrome P450 enzyme subfamily, thus forming 25-hydroxyvitamin D_3_ or calcifediol (25(OH)D_3_) [5]. This compound shows an average plasma life of about three weeks, and therefore its serum levels are now currently used as indicators of the body vitamin D storage and status of patients. However, calcifediol is metabolically inactive and, once produced, it is secreted into blood bound up with vitamin D binding protein (DBP). In this case, the calcifediol needs a renal 1α-hydroxylation to obtain the biologically active form of vitamin D_3_, 1α,25(OH)_2_D_3_ [1] (Figure 1).

Vitamin D deficiency has been observed in different subpopulations, suggesting that it is an essential nutrient for people from certain ethnic groups, as well as people with specific lifestyle behaviors, such as elderly people, those who do not get enough sunlight exposure, and individuals with darker skin pigmentation [6]. Moreover, the relationship between the vitamin D status and the occurrence of several chronic illnesses (e.g., diabetes and cancer) proves that the physiological actions of this hormone go beyond the traditional effect on calcium homeostasis maintenance [7]. The presence of 1α-hydroxylases isoforms in extrarenal tissues implies that calcitriol could act as a paracrine or autocrine signal as well as its classical endocrine role [8].

Calcitriol acts through the binding with a VDR, which has been described in different species including humans, rats, and chickens [9]. It has a ligand-binding domain, named E-domain, a DNA-binding domain named C-domain, and an F-domain, which is one of the active domains. It acts by binding vitamin D-responsive elements (VDREs), repeated sequences located in the proximity of the start site of the target gene [10]. Upon the interaction between the ligand and the VDR, there is a conformational rearrangement of the receptor, preventing the bonding of the repressor. This results in the formation of a heterodimer between the VDR and the retinoid X receptor (RXR) at the VDREs, thus either initiating or suppressing the gene transcription whose protein products are involved in the controlling of calcium homeostasis (i.e., cytochrome P450 family 24 (CYP24), alkaline phosphatase (ALP), type I collagen (COL1A1), osteocalcin (bone gamma-carboxyglutamate protein, BGLAP), and transient receptor potential vanilloid type family member 6 (TRPV6)) [1,11,12]. Since VDR has been found in nearly all cell types, this could clarify the different vitamin D_3_ actions on several types of tissues [11].

In these circumstances, the increase or reduction of protein expression levels through gene transcription regulation from steroid hormones is the consequence of genomic steroid action [13]. These actions are not acute but delayed, because they require time for newly synthesized proteins and their processing [13]. In addition, molecules inhibiting of transcription and protein synthesis, such as cycloheximide or actinomycin D, may completely block these delayed effects if they do not take place at the time when steroid molecules are coupled to large proteins, thus preventing their cell entry [13].

Since the final active form was isolated and identified in 1971 [14], additional noncalcemic functions in the body have been described through further investigation of the importance of this hormone in the endocrine system.

In recent years, calcitriol, as well as its direct precursor, calcifediol, have been shown to exert rapid non-genomic steroid actions, indicating a more complex mechanism responsible for the wide range of actions of vitamin D [15,16].

The aim of this non-systematic is to provide an overview of the rapid, non-genomic effects of calcifediol and calcitriol, focusing on the mechanisms underlying these rapid responses that could lead to a better understanding of the vitamin D_3_ endocrine system, thus paving the way for the identification of potential novel therapeutic options for pathological conditions associated with vitamin D_3_ deficiency.

For this purpose, a rigorous search for literature on PubMed database has been conducted by employing different combinations of relevant keywords, including “vitamin D”, “1α,25(OH)_2_D_3_”, “25(OH)D_3_”, “non-genomic effects”, “VDR”, and “Pdia3”. All relevant studies released were selected and reviewed.

## 2. Rapid, Non-Genomic Steroid Actions

The first non-genomic action of steroid molecules was described in 1942 when Hans Selye observed the anesthetic effect of progesterone immediately after its injection into peritoneum of rats and mice differently from what was observed with respect to its main effect that took place only within hours after its administration [17]. Subsequently, Spach and Streeten demonstrated that Na^+^ ion variation occurred within few minutes after aldosterone administration in dog erythrocytes, offering new compelling evidence on non-genomic effects of this hormone because these cells lack nuclei, and therefore the observed in vitro effects might be attributable exclusively to its non-genomic mechanism [18]. However, these rapid hormone effects were not clarified until their recent recognition for several steroid hormones, including 1α,25(OH)_2_D_3_ [13].

Recently, 25(OH)D_3_, for a considerable time deemed only a metabolic precursor of 1α,25-(OH)_2_D_3_, has been proved to be an agonist ligand of VDR and capable of initiating rapid, non-genomic actions [16,19].

As opposed to their genomic counterpart, non-genomic rapid responses appear rapidly (within a range of seconds or minutes), are not susceptible to cycloheximide or actinomycin D, and also occur in response to steroids coupled to macromolecules which block their cell entering [20]. Therefore, a major difference for discerning between genomic and non-genomic actions is the time course and the sensitivity of transcription and protein synthesis inhibitors.

## 3. Mechanisms of Membrane-Associated Proteins for 1α,25(OH)_2_D_3_-Mediated Rapid, Non-Genomic Actions

Concerning the existence of non-genomic calcitriol actions, to our knowledge, the earliest observations for these actions were described by Nemere et al. in 1984 [21], who observed that this hormone was able to induce a rapid increase of intracellular calcium (Ca^2+^) concentrations both by promoting its release from intracellular stores and by stimulating its intestinal uptake in the vascularly perfused duodenum of normal, vitamin D-replete chicks. They observed that calcitriol significantly increased Ca^2+^ transport within 14 min compared with controls in a mechanism independent of genome activation and de novo protein synthesis. This rapid effect on transepithelial Ca^2+^ movement across the intestine has been termed transcaltachia.

It is established that the biologically active vitamin D_3_ metabolite stimulates different signaling molecules, including phospholipase A2 (PLA2), phospholipase C (PLC), and phosphatidylinositol-3 kinase (PI3K), and promotes the generation of second messengers, such as Ca^2+^ ions, phosphatidylinositol (3,4,5)-trisphosphate (PIP3), and cyclic AMP (cAMP), culminating in the activation of several downstream protein kinases (protein kinase C (PKC), calcium/calmodulin-dependent protein kinase II gamma (CaMKIIG), Src, and mitogen-activated protein (MAP) kinases) [22,23,24,25,26]. In addition to the above-mentioned non-genomic actions of 1α,25(OH)_2_D_3_, this secosteroid hormone also mediates the opening of Ca^2+^, Cl^−^, and Pi channels.

Early studies performed by Norman and colleagues [27,28] suggested that a distinct membrane VDR could mediate non-genomic actions in response to 1α,25(OH)_2_D_3_. In their study, the authors reported that the biologically active form of vitamin D_3_ promotes the generation of non-genomic responses and, especially in the case of transcaltachia, in its 6-s-cis configuration. By contrast, binding of secosteroid in the 6-s-trans form could be responsible for genomic responses.

Further studies have shown that membrane-associated VDR (VDRm) could bind to proto-oncogene, non-receptor tyrosine kinase Src, and caveolin 1 (CAV1) in caveolae-enriched plasma membranes, thereby participating in the regulation of many signaling pathways, such as sonic hedgehog (Shh) [29,30,31,32,33,34], Wnt [35,36,37,38], and Notch [39,40,41].

In 1990, Civitelli et al. [42] observed an acute and transient rise in calcium mobilization in osteoblastic osteosarcoma cell line ROS 17/2.8 after a 1α,25(OH)_2_D_3_ stimulation in a mechanism independent of genomic activation, through both the influx of extracellular Ca^2+^ and release of Ca^2+^ from intracellular stores. The active form of vitamin D_3_ also produces a significant increase in the production of diacylglycerol (DAG) and inositol 1,4,5-trisphosphate (IP3), resulting in the activation of PLC.

This rapid effect was also observed in primary muscle cell cultures isolated from a chicken embryonic heart [43]. In particular, 1α,25(OH)_2_D_3_ induces a fast increase of both tissue Ca^2+^ uptake and cAMP levels within 10 min in primary myocytes. Moreover, authors found that this effect was inhibited by a specific protein kinase A (PKA) suppressor, suggesting that the regulation of Ca^2+^ ion channel activity by the biologically active form of vitamin D_3_ was mediated by the second messenger cAMP.

Baran et al. [44] demonstrated that the secosteroid 1α,25(OH)_2_D_3_ not only evokes a rapid opening of Ca^2+^ channels but also a rapid activation of phospholipase C (PLC) in ROS 24/1 osteoblastic cells lacking the VDR, implying that these effects occur independently of the VDR signaling mechanisms. This observation was also confirmed in vivo, where Boyan et al. [45] observed that 1α,25(OH)_2_D_3_ is involved in the regulation of protein kinase C (PKC) activity through rapid membrane-associated mechanisms in cultured costochondral chondrocytes derived from VDR^−/−^ mice.

This could suggest that, in addition to membrane-associated VDR, the existence of other membrane receptors in conjunction with vitamin D might be essential for rapid, non-genomic effects of 1α,25(OH)_2_D_3_.

Given the highly liposoluble nature of vitamin D_3_, 1α,25(OH)_2_D_3_, can penetrate biological membranes, and when inside the cells, may interact with heat shock proteins (HSPs) so as to be transferred to the nucleus and the mitochondria [46]. Alternatively, vitamin D_3_ metabolites bound to a specific vitamin D_3_ binding protein (DBP) can undergo endocytosis through an LDL receptor-related protein 2 (LRP2)- and cubilin (CUBN)-mediated mechanism. Subsequently, 25(OH)D_3_ bound to DBP is transported into the proximal tubule epithelium of the kidneys via megalin-mediated endocytosis, where it undergoes a hydroxylation step to transform into 1α,25(OH)_2_D_3_ [47,48]. In this light, additional studies should be performed to clarify the physiological significance and the potential mechanistic aspect for vitamin D_3_ transporters.

One of best membrane-associated proteins able to bind vitamin D_3_ compounds is the protein disulfide isomerase family A member 3 (Pdia3), also known as 1α,25(OH)_2_D_3_-membrane-associated rapid response to steroid (MAARS), which has been described as a crucial protein in 1α,25(OH)_2_D_3_-initiated rapid membrane non-genomic signaling pathways [49].

This protein was first purified in the study of Nemere et al. [50], describing the existence of a putative plasmalemmal receptor for the biologically active form of vitamin D_3_ involved in the transcaltachia on the basal-lateral membranes (BLM) of chick intestinal epithelium. This conclusion was supported by the observation that an altered, but still present, specific binding for [^3^H] 1α,25(OH)_2_D_3_ was discovered in membrane fractions purified from vitamin D-deficient chicks with respect to the corresponding fractions obtained from normal animals. In addition, the BLM-VDR exhibited down-regulation of specific [^3^H] 1α,25(OH)_2_D_3_ binding upon exposure to nonradioactive 1α,25(OH)_2_D_3_.

Later studies showed that this candidate plasmalemmal receptor essential for the rapid responses by 1α,25(OH)_2_D_3_ is Pdia3 [51,52]. Its primary function is to catalyze the formation, reduction, and isomerization of disulfide bonds, interacting with lectin-like molecular chaperones, calnexin (CANX) and calreticulin (CALR), to ensure the correct folding of newly synthesized glycoproteins [53].

Pdia3 has been identified in the cell membrane, cytosol, mitochondria, and the nucleus and has demonstrated multiple distinct functions, such as the protection against oxidative stress and the prevention of diseases associated with the accumulation of unfolded/misfolded proteins [54].

One remarkable function of Pdia3 is its involvement in one of the most prominent rapid actions of 1α,25(OH)_2_D_3_, namely transcaltachia. Importantly, it has been observed that the rapid increase in intracellular Ca^2+^ levels in response to subnanomolar concentrations of 1α,25(OH)_2_D_3_ involves the interaction between Pdia3 and CAV1, the principal component of the caveolae plasma membranes and small plasma membrane invaginations [23,55,56].

Moreover, the interaction between the biologically active form of vitamin D_3_ and Pdia3 also has a significant implication for the cell protection against UV-caused thymine dimer formation [57], the activation of PKC signaling transduction pathway [58], and the repression of tumor necrosis factor receptor signaling produced by a rapid rise in intracellular Ca^2+^ concentration in aortic smooth muscle cells [59].

Interestingly, further studies have shown that Pdia3 protein binding of 1α,25(OH)_2_D_3_ is also involved in the activation of PLA2 via PLA2 activating protein (PLAA) [49], MAPK1, and MAPK2 via the regulation of CaMKIIG, PLC, PLA2, and PKC [23,55], and Wnt family member 5A (Wnt5A) [60].

Collectively, the above-mentioned studies investigating rapid, non-genomic actions in response to 1α,25(OH)_2_D_3_ support the hypothesis that Pdia3 is a crucial component of the machinery responsible for mediating these vitamin D_3_ activities.

Consistent with data derived from in vitro studies, it has been reported that Pdia3 is involved in vitamin D-mediated actions in vivo. Despite the observation of the fact that homozygous Pdia3 deletion causes embryonic lethality in mice, the animals with a deleted allele develop skeletal abnormalities, thus suggesting its function in maintaining calcium homeostasis [61,62]. In this regard, an analysis of enterocytes isolated from Pdia3 knockout mice showed a reduction in rapid, non-genomic effects following 1α,25(OH)_2_D_3_, such as the PKA signaling pathway and calcium uptake [63]. Moreover, the loss of Pdia3 in deficient mice resulted in significant attenuation of 1α,25(OH)_2_D_3_-related PKC activation and skeletal abnormalities [61]. Importantly, a study by Boyan et al. revealed that cultured chondrocytes isolated from VDR knockout mice retaining Pdia3 gene expression showed a rapid increase in PKC activity after 1α,25(OH)_2_D_3_ treatment [45].

Based on this evidence, Pdia3 could be involved in the activation of rapid non-genomic actions mediated by 1α,25(OH)_2_D_3_, which in turn might have a significant impact on musculoskeletal biology regulation, including calcium absorption through the intestinal epithelial and skeletal development.

However, crystallographic studies have not confirmed any binding site for the biologically active form of vitamin D_3_ in the partial structure of Pdia3 [64], although it has been assumed that its activity could be essential for the rapid, non-genomic actions of this secosteroid hormone. In this respect, not only was it postulated that 1α,25(OH)_2_D_3_ could interact with Pdia3 through its a’ domain, which has been shown to be essential for triggering non-genomic actions, but also that Pdia3 could function as a molecular chaperone for VDR, DPB, or other unknown membrane-associated proteins [64,65].

In Table 1, we summarized the 1α,25(OH)_2_D_3_-mediated rapid, non-genomic actions.

## 4. 25(OH)D_3_-Mediated Rapid, Non-Genomic Actions

In the study conducted by Lou et al. [19], it was assumed that calcifediol is an agonist ligand of VDR with anti-proliferative effects and gene regulatory functions, despite the fact it binds to VDR with a lower affinity compared to the biologically active metabolite of vitamin D_3_.

Based on this interesting observation, our previous research article [16] aimed to evaluate whether this metabolite could initiate rapid, non-genomic pathways, such as an increase in intracellular Ca^2+^ concentrations, in line with what was established for calcitriol. We observed for the first time that calcifediol produces a rapid increase in Ca^2+^ levels in mesenchymal stem cells derived from human adipose tissue (hADMSCs), although at a higher concentration compared to those present in normal physiological conditions. In this regard, calcifediol at subnanomolar concentrations has been revealed incapable of triggering an increase of intracellular Ca^2+^ concentration in human spermatozoa although an evident but delayed effect was found at higher dose [66,67].

Another study by Asano et al. showed an interesting non-genomic mechanism of 25(OH)D_3_, where this compound could regulate lipogenesis, thereby reducing the risk of metabolic disease-associated complications by altering sterol regulatory element-binding proteins (SREBPs) activation via the ubiquitin-mediated proteasomal degradation of SREBP cleavage-activating protein (SCAP) [68].

In Table 2, we summarized the 25(OH)D_3_-mediated rapid, non-genomic actions.

## 5. Discussion

In recent years, 1α,25(OH)_2_D_3_, the biologically active metabolite of vitamin D_3_, has attracted attention due to its involvement in several biological processes, including the regulation of the serum levels of calcium and phosphate, as well as its influence on bone and mineral metabolism.

There is now compelling evidence that 1α,25(OH)_2_D_3_ affects the target cells through genomic pathways and membrane receptor-mediated rapid, non-genomic responses. This latter mechanism has also been described for virtually all the steroid molecules, such as aldosterone, testosterone, estrogens, and cortisol [16,49,69].

Recently, even the direct metabolic precursor of 1α,25(OH)_2_D_3_, named calcifediol, it has been revealed able to activate rapid, nontranscriptional actions, such as an acute and sustained rise in intracellular Ca^2+^ levels, similarly to that observed with the biologically active form of vitamin D_3_ [16,70].

As described in this review, although growing evidence has led to a significant knowledge concerning calcifediol and calcitriol rapid, non-genomic activities, their impact on physiological processes needs to be clarified. In fact, it is difficult to identify vitamin D-deficiency-associated diseases which occur exclusively due to aberrations of its rapid actions.

The vitamin D_3_ endocrine system is primarily involved in different biological processes that maintain calcium and bone homeostasis, and presumably, it could not benefit from rapid membrane-mediated non-genomic responses. However, these actions could have important physiological implications for some processes, in particular on the protection of cells against DNA damage caused by solar UV radiation exposure and intestinal Ca^2+^ absorption.

In this regard, one of the most noticeable rapid actions of 1α,25(OH)_2_D_3_ is transcaltachia, the rapid stimulation of intestinal Ca^2+^ transport. However, further studies are needed to link this rapid physiological manifestation of 1α,25(OH)_2_D_3_ with meal feeding and therefore with calcium absorption physiology.

Accumulating evidence has proposed that the rapid, non-genomic actions could positively or negatively affect the genomic function mediated by 1α,25(OH)_2_D_3_ [15]. In fact, the secosteroid hormone could activate various signaling molecules involved in several rapid transduction pathways (i.e., PKC, PI3K, and PLA2) which could affect gene expression either through transcriptional regulatory elements present in the promoter or by using activated VDR as a substrate [15,71]. Moreover, this cross-talk could regulate both the efficacy and potency of genomic function [71].

Although the subject of vitamin D-mediated rapid non-genomic effects has been explored in recent years, important questions should be taken into account in the foreseeable future.

The current data come from a combination of results of studies performed on different species and multiple cells. Since these actions could be dependent on cell types, cell cycle stage and species, future studies should be carried out to elucidate the mechanisms underpinning these effects in a wide range of cells.

In addition, it should be considered that most of the in vitro studies about non-genomic effects of vitamin D_3_ compounds use higher concentrations than those subnanomolar existing under normal physiological circumstances [16,49,66]. This finding could be because secosteroid-related rapid, non-genomic actions could require higher concentrations than the genomic counterpart.

It has now been established that the VDR is merely the only protein that binds with the active form of vitamin D_3_ at a high level of affinity. Moreover, it is scattered in more than 38 tissues; this evidence could explain the diversity of actions on different tissues [1,71].

The vitamin D-endocrine system is complex, and there are several conversion and transport processes necessary to produce compounds in the skin by UV radiation until the synthesis of the active metabolite of vitamin D_3_ is reached.

During its production, besides undergoing the action of specific enzymes, vitamin D_3_ compounds interact with not only VDR but also other binding/transport proteins, such as DPB in the circulation and heat shock protein family A (HSPA) when inside the cells.

A growing body of evidence suggests that Pdia3, the best outlined membrane-associated protein capable of binding with vitamin D_3_ compounds, could play a key role in 1α,25(OH)_2_D_3_-mediated non-genomic responses [23,55,56]. Although studies regarding the crystal structure of Pdia3 did not reveal any binding pocket for the secosteroid hormone, its activity is critical for the rapid actions of 1α,25(OH)_2_D_3_. In this respect, even though it might not bind directly 1α,25(OH)_2_D_3_, this protein could serve as a molecular chaperone for DBP or VDR [49].

However, little is known about the presence of other nonclassical membrane receptors that stand at the beginning of membrane-based rapid responses to vitamin D_3_ and its metabolites as well as the existence of alternative cell signalling transduction pathways. Their comprehension could help in understanding of the non-genomic effects of vitamin D that have not been settled yet. 

**Figure 2:**
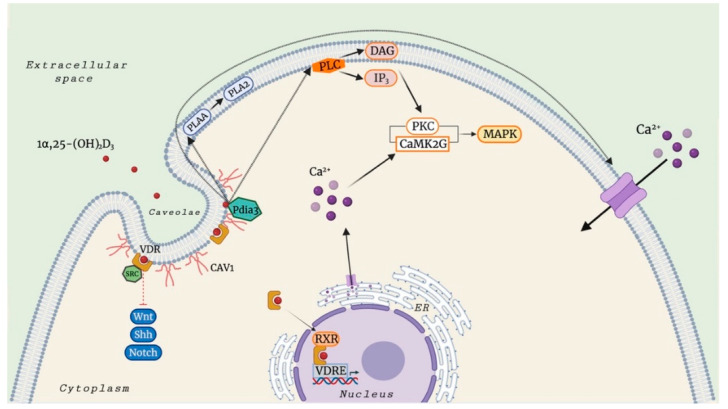
The proposed genomic and non-genomic mechanisms of the biological active form of vitamin D_3_, 1α,25(OH)_2_D_3_. Abbreviations: VDR: vitamin D_3_ receptor; RXR: retinoid X receptor; VDRE: vitamin D_3_ response elements; CAV1: caveolin 1; Shh: Sonic hedgehog; Pdia3: protein disulphide isomerase family A member 3; PLA2: phospholipase A2; PLAA: PLA2 activating protein; PLC: phospholipase C; DAG: diacylglycerol; IP_3_: inositol trisphosphate; PKC: protein kinase C; CaMK2G: calcium/calmodulin-dependent protein kinase II gamma; MAPK: mitogen-activated protein kinase; SRC: SRC proto-oncogene, non-receptor tyrosine kinase; Wnt: Wingless/Integrated. Image created by BioRender (https://app.biorender.com (accessed on 14 February 2022)).

In summary, a significant progress has been made in this field in recent years, shifting the understanding of vitamin D_3_ beyond the originally reported role in calcium homeostasis and prevention of rickets in children and osteomalacia in adults. It has now been established not only that the vitamin D-related action mechanism is not based solely on its genomic activity, but also that either calcitriol or calcifediol can promote the generation of rapid, non-genomic effects, as well as described for other steroid hormones that can impact different physiological processes. There is convincing evidence that VDR has an essential role in these rapid responses. As described in this review, other proteins, such as Pdia3, may also bind to 1α,25(OH)_2_D_3_, although with a lower affinity compared to the VDR, thereby playing a crucial role in rapid membrane non-genomic response to vitamin D_3_ (Figure 2).

Further studies will need to be performed to better understand the mode of action sustaining these rapid responses to provide new avenues for the development of novel therapeutic approaches able to modulate the non-genomic actions of vitamin D, especially for people who are vitamin D_3_-deficient.

In fact, there are limited human data available for the non-genomic actions of vitamin D in vivo after a dietary supplementation and therefore additional investigations are required to elucidate whether vitamin D_3_ metabolites could elicit the same rapid nontranscriptional effects that have been reported under in vitro conditions. 

## Figures and Tables

**Table 1:** Overview of the 1α,25(OH)_2_D_3_-mediated rapid, non-genomic actions.

Study	1α,25(OH)_2_D_3_-Mediated Rapid, Non-Genomic Effects	Putative Membrane-Associated Protein Responsible for 1α,25(OH)_2_D_3_-Related Rapid, Non-Genomic Effects
Nemere et al. [21]	Transcaltachia	/ ^1^
Dormanen et al. [27,28]	Transcaltachia	VDR
Lisse et al. [32] and Teichert et al. [33,34]	Regulation of the Hedeghog signalling pathway	VDR
Tapia et al. [35], Muralidhar et al. [36], and Tang et al. [37]	Regulation of the Wnt signalling pathway	VDR
Wang et al. [39] and Olsson et al. [40]	Regulation of the Notch signalling pathway	VDR
Civitelli et al. [42]	Increase in intracellular Ca^2+^ levels	/ ^1^
Selles et al. [43]	Involvement in cAMP signalling pathway	/ ^1^
Nemere et al. [50]	Transcaltachia	Pdia3
Doroudi et al. [56]	Increase of intracellular Ca^2+^ levels	Pdia3
Sequeira et al. [57]	Protection of UV-induced thymine dimer formation	Pdia3
Khanal et. al. [58]	Regulation of PKC signalling pathway	Pdia3
Yang et al. [59]	Regulation of TNF signalling pathway	Pdia3
Zmijewski et al. [49]	Regulation of PLA2 activation	Pdia3
Doroudi et al. [55]	Regulation of MAPK1 and MAPK2 activation	Pdia3
Doroudi et al. [60]	Regulation of Wnt5A non-canonical signalling pathway	Pdia3
Nemere et al. [63]	Regulation of PKA signalling pathway	Pdia3
Wang et al. [61] and Boyan et al. [45]	Regulation of PKC activity	Pdia3

/ ^1^ Data not reported. VDR: vitamin D receptor; Pdia3: protein disulfide isomerase family A member 3; Wnt: Wingless/Integrated; cAMP: cyclic adenosine monophosphate; PKC: protein kinase C; TNF: tumor necrosis factor; PLA2: phospholipase A2; MAPK: mitogen-activated protein kinase; PKA: protein kinase A.

**Table 2:** Overview of the 25(OH)D_3_-mediated rapid, non-genomic actions.

Study	25(OH)D_3_-Mediated Rapid, Non-Genomic Effects	Putative Membrane-Associated Protein Responsible for 25(OH)D_3_-Related Rapid, Non-Genomic Effects
Donati et al. [16]	Increase of intracellular Ca^2+^ levels	/ ^1^
Jensen et al. [67]	Increase of intracellular Ca^2+^ levels	VDR
Asano et al. [68]	Regulation of lipogenesis	SCAP

/ ^1^ Data not reported. VDR: vitamin D receptor; SCAP: SREBP cleavage-activating protein.

## Data Availability

Not applicable.
